# Mapping the binding site of the P2X receptor antagonist PPADS reveals the importance of orthosteric site charge and the cysteine-rich head region

**DOI:** 10.1074/jbc.RA118.003737

**Published:** 2018-07-11

**Authors:** Hong Huo, Alistair G. Fryatt, Louise K. Farmer, Ralf Schmid, Richard J. Evans

**Affiliations:** From the ‡ Department of Molecular and Cell Biology and; § Leicester Institute of Structural and Chemical Biology, University of Leicester, Leicester, United Kingdom

**Keywords:** purinergic receptor, mutagenesis, ion channel, oocyte, molecular docking, antagonist, cysteine, orthosteric site, P2XR, PPADS

## Abstract

ATP is the native agonist for cell-surface ligand-gated P2X receptor (P2XR) cation channels. The seven mammalian subunits (P2X1–7) form homo- and heterotrimeric P2XRs having significant physiological and pathophysiological roles. Pyridoxalphosphate-6-azophenyl-2′,4′-disulfonic acid (PPADS) is an effective antagonist at most mammalian P2XRs. Lys-249 in the extracellular domain of P2XR has previously been shown to contribute to PPADS action. To map this antagonist site, we generated human P2X1R cysteine substitutions within a circle centered at Lys-249 (with a radius of 13 Å equal to the length of PPADS). We hypothesized that cysteine substitutions of residues involved in PPADS binding would (i) reduce cysteine accessibility (measured by MTSEA-biotinylation), (ii) exhibit altered PPADS affinity, and (iii) quench the fluorescence of cysteine residues modified with MTS-TAMRA. Of the 26 residues tested, these criteria were met by only four (Lys-70, Asp-170, Lys-190, and Lys-249), defining the antagonist site, validating molecular docking results, and thereby providing the first experimentally supported model of PPADS binding. This binding site overlapped with the ATP-binding site, indicating that PPADS sterically blocks agonist access. Moreover, PPADS induced a conformational change at the cysteine-rich head (CRH) region adjacent to the orthosteric ATP-binding pocket. The importance of this movement was confirmed by demonstrating that substitution introducing positive charge present in the CRH of the hP2X1R causes PPADS sensitivity at the normally insensitive rat P2X4R. This study provides a template for developing P2XR subtype selectivity based on the differences among the mammalian subunits around the orthosteric P2XR-binding site and the CRH.

## Introduction

P2X receptors (P2XRs)^2^ comprise a family of cation channels that are gated by the binding of extracellular ATP (1). The seven P2XR subunits (P2X1–7) assemble to form homo- and heterotrimeric receptors with a range of properties (2). Analysis of the expression patterns of P2XR subunits suggests that at least one receptor subtype is expressed by almost every cell type at some time (3). Distinct roles of defined subunits in a panoply of physiological and pathophysiological conditions have been described. For example, P2X1Rs contribute to neurogenic control of smooth muscle contraction and P2X3Rs mediate pain sensation (for review, see Ref. 1). One important tool in determining the functional contribution of P2XRs has been the sensitivity of responses to the P2X receptor antagonist pyridoxalphosphate-6-azophenyl-2′,4′-disulfonic acid (PPADS) (1, 4).

PPADS was first described as a P2XR antagonist in studies on native smooth muscle preparations (5) that express functional P2X1Rs (6). Subsequently PPADS was shown to be effective at other native and recombinant P2XRs (1). At human P2XRs sensitivity to PPADS depended on the subtype and was highest at the hP2X1, -2, -3, -5, and -7Rs with an IC_50_ of ∼1–3 and ∼30 μm for the hP2X4R (1). PPADS is a negatively charged molecule comprising a phosphate and two sulfonate groups. The first clue as to the potential site of action of PPADS came from studies on the antagonist-insensitive rat P2X4R. These showed that mutation of the negatively charged glutamate residue Glu-249 to lysine, which is found in PPADS sensitive P2X1 and -2Rs, introduced PPADS sensitivity (7). Subsequently the generation of chimeric rat/human P2X4Rs suggested that variations in the stretch of residues between 102 and 176 (22 amino acid differences), which incorporates the cysteine-rich head (CRH) region, also contribute to PPADS action (8). At the P2X7R, where the sensitivity to PPADS is species dependent, the R126G mutant (human to rat) at the base of the CRH region decreased PPADS affinity ∼10-fold (9). The mutants E249K (rP2X4R) and R126G (hP2X7R) are located on the periphery of the ATP-binding pocket suggesting that PPADS binding blocks access to the orthosteric site.

Recent crystallographic studies have identified the binding sites of subtype selective antagonists for P2X3 and -7Rs (10–12). However, no structural information is currently available for PPADS. In this study we have used a combination of biochemical, electrophysiological, imaging, and modeling approaches to identify the PPADS-binding site. Based on predictions from our data, we were able to introduce PPADS sensitivity to the rP2X4R.

## Results

### Mapping changes in accessibility of cysteine mutants in response to PPADS binding

**Figure 1.**
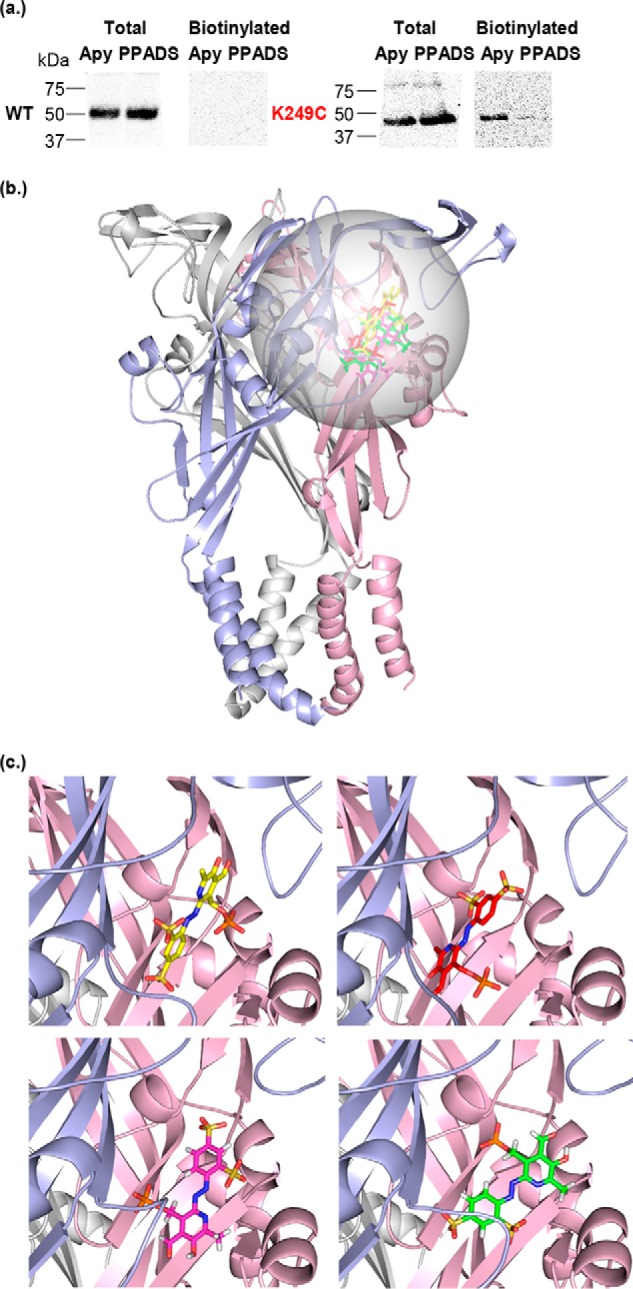
**Access to the K249C hP2X1R mutant is reduced by PPADS binding and was the starting point for RosettaLigand docking.** *a*, representative blots of MTSEA-biotinylation of hP2X1 receptor WT and the K249C mutant. Oocytes were treated with apyrase (*Apy*, 15 units/ml) or apyrase + PPADS (100 μm). Data show expression of the P2X1 in the total samples, however, biotinylation of the WT receptor was below the limit of detection. The K249C mutant receptor was biotinylated and this was reduced by PPADS treatment. *b*, PPADS docked into both, apo and open state hP2X1R models (for clarity only the open state receptor is shown). Individual subunits are colored in *blue*, *pink*, and *gray*. Docking was focused on the extracellular region centered at Lys-249, the sampled space is indicated by a *transparent sphere*. Representative docked ligands of the top ranking clusters for open and apo states are shown as *sticks. c*, zoom into *b*, representative docking solutions of top ranking clusters for hP2X1R apo state models (*bottom*) and hP2X1R open state models (*top*). These clusters were further analyzed for agreement/disagreement with the experimental data presented in this work.

**Figure 2.**
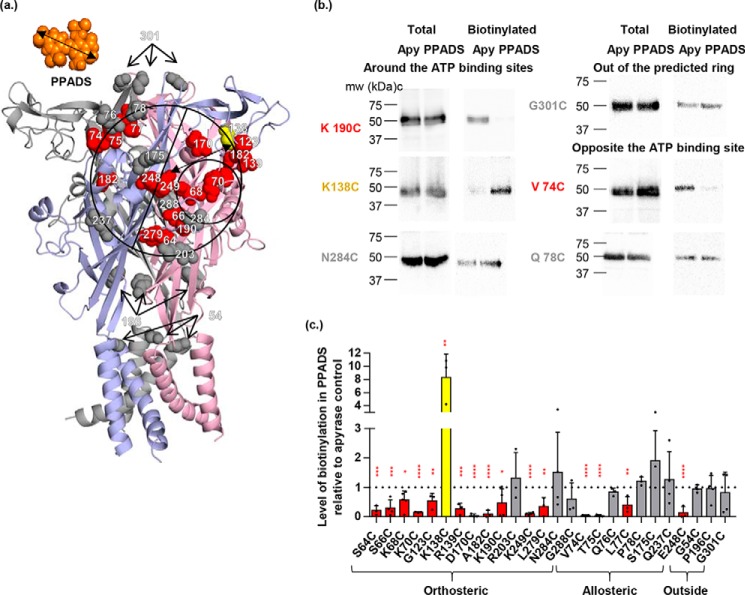
**Effect of PPADS on accessibility changes of the cysteine mutants in the hP2X1 receptor.** *a,* the closed state homology model of hP2X1 receptor is shown in *cartoon*, with three subunits in *blue*, *pink*, and *gray,* respectively. The *black ring* is centered on residue 249 with a radius the length of PPADS, the *circle* can be divided by the line into two parts (around the ATP-binding sites shown as a smaller circle, and opposite to the ATP-binding site). Cysteine-mutated residues are shown as *spheres* (*red*, decreased accessibility in the presence of PPADS, *yellow*, increased accessibility, *gray*, no significant change). *b*, representative blots of MTSEA-biotinylation of hP2X1 receptor mutants. Oocytes were treated with apyrase (*Apy*, 15 units/ml) or apyrase + PPADS (100 μm). *c*, densitometric analysis using ImageJ showing any change in MTSEA-biotinylation level at each individual cysteine mutant in the presence of PPADS compared with the apyrase control (*n* = 3), data are shown as mean ± S.D., (*n* > 3) (*, *p* < 0.05; **, *p* < 0.01; ***, *p* < 0.001).

**Figure 3.**
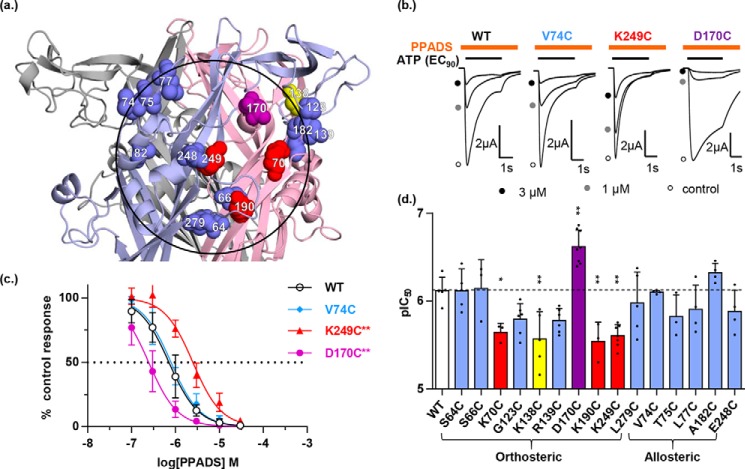
**Effect of individual cysteine mutants on PPADS sensitivity of the hP2X1 receptor.** *a,* the closed state homology model of hP2X1R zoomed in on the extracellular loop showing the location of individual cysteine mutants, *blue spheres* indicate decreased accessibility but no change in PPADS potency, *red spheres* indicate decreased accessibility and PPADS potency, *yellow spheres* indicate increased accessibility and decreased PPADS potency, *purple sphere* indicates decreased accessibility and increased PPADS potency. The *black ring* is centered on residue 249 with a radius of the length of PPADS. *b*, representative traces of inhibition of ATP-evoked currents in the absence (control) and presence of PPADS. Different concentrations of PPADS were applied 5 min before the co-application with ATP (EC_90_). *Orange bar* indicates application of PPADS and *black bar* indicates application of ATP. *c*, PPADS concentration-dependent inhibition curves of the hP2X1R mutants V74C, K249C, and D170C. The WT receptor response is shown with as a *black line* (*n* = 3). *d*, comparison of PPADS potency (pIC_50_) between WT and mutant hP2X1Rs. Statistical analysis was performed by one-way analysis of variance, data are shown as mean ± S.D., *n* = 3 (*, *p* < 0.05; **, *p* < 0.01; ***, *p* < 0.001).

We have previously used cysteine mutagenesis and MTSEA-biotinylation at hP2X1Rs to determine whether a defined residue was (i) accessible at the receptor extracellular surface and (ii) contributed to ligand action (13, 14). We posited that if a residue was involved in PPADS binding that the antagonist would reduce accessibility of an introduced cysteine at that position and that the mutation would also have an effect on antagonist sensitivity. The starting point of our study was the observation by Buell *et al.* (7) on the rP2X4R showing that replacing a negatively charged glutamic acid residue with a positively charged lysine residue (found in P2X1 and -2Rs) at position 249 introduced PPADS sensitivity. We were therefore interested to see if the corresponding lysine residue in the hP2X1R (Lys-249) directly contributed to PPADS binding. The cysteine mutation of this residue in the hP2X1R (K249C) had no effect on ATP sensitivity or peak current amplitude compared with WT hP2X1Rs (Table 1). At WT hP2X1Rs following MTSEA-biotin treatment no biotinylation of the P2X1R was detected (the receptor was present in the total oocyte lysate sample, Fig. 1) consistent with our previous studies demonstrating the lack of free cysteine residues in the WT receptor; the 10 conserved cysteine residues in the extracellular loop form five disulfide bonds and the only other cysteine in the hP2X1R is in the second transmembrane segment and so inaccessible to membrane impermeant MTSEA-biotin (13–15). In contrast, the introduced cysteine at position 249 was labeled with MTSEA-biotin under control conditions (in the presence of apyrase to break down any endogenous ATP) and this labeling was inhibited by >90% following PPADS treatment (100 μm for 10 min) (Figs. 1 and 2). This demonstrates that accessibility to residue 249 was reduced by PPADS binding. If this results from Lys-249 contributing to the PPADS-binding site we would predict that removal of the positively charged lysine would decrease antagonist affinity. This was the case with PPADS sensitivity reduced ∼3-fold from 0.8 to 2.5 μm (pIC_50_ values of 6.12 ± 0.15 and 5.60 ± 0.05 for WT and K249C, respectively, *p* < 0.01, Fig. 3). Taken together the reduction in accessibility to K249C in the presence of PPADS and the reduction in PPADS sensitivity strongly suggests that residue 249 is part of the binding site for the antagonist.

**Table 1:** **Properties of wildtype and mutant P2X1Rs that showed a change in MTSEA-biotinylation in response to PPADS treatment** Peak currents to a maximal concentration of ATP and the sensitivity to ATP (pEC_50_), the EC_90_ concentration used for determination of antagonist sensitivity and PPADS (pIC_50_); data are shown as mean ± S.D.

Mutants	Peak	*n*	pEC_50_	EC_50_	*n*	EC_90_	pIC_50_	IC_50_	*n*
	μ*A*			μ*m*		μ*m*		μ*m*	
WT	−10.06 ± 3.58	37	6.12 ± 0.16	0.8	4	10	6.12 ± 0.15	0.8	5
S64C	−13.47 ± 0.96	5	5.90 ± 0.11	1.2	3	10	6.12 ± 0.25	0.8	4
S66C	−11.56 ± 0.73	4	5.80 ± 0.11	1.6	3	10	6.14 ± 0.33	0.7	3
K70C	−**4.21 ± 0.88*^a^***	3	**4.34 ± 0.16*^b^***	**45.9**	3	**300**	**5.64 ± 0.10*^a^***	**2.3**	3
V74C	−10.46 ± 0.81	6	6.02 ± 0.10	1.0	3	10	6.10 ± 0.02	0.8	4
T75C	−7.60 ± 2.57	4	**5.60 ± 0.05*^c^***	**2.5**	3	**10**	5.82 ± 0.25	1.5	3
L77C	−11.23 ± 2.34	6	5.93 ± 0.09	1.2	3	10	5.90 ± 0.28	1.3	4
G123C	−6.61 ± 4.63	6	**5.21 ± 0.07*^b^***	**6.1**	4	**30**	5.79 ± 0.18	1.6	6
K138C	−**14.47 ± 2.42*^a^***	5	6.34 ± 0.09	0.5	3	10	**5.56 ± 0.31*^b^***	**2.8**	5
R139C	−**16.96 ± 1.16*^b^***	5	6.22 ± 0.07	0.6	3	10	5.78 ± 0.14	1.7	6
D170C	−10.90 ± 0.2	5	**5.69 ± 0.14*^c^***	**2.1**	5	**10**	**6.62 ± 0.19*^d^***	**0.2**	8
A182C	−7.24 ± 2.11	4	6.26 ± 0.25	0.6	3	10	6.32 ± 0.11	0.5	4
K190C	−**5.39 ± 0.49*^a^***	4	**5.46 ± 0.06*^b^***	**3.5**	3	**30**	**5.54 ± 0.22*^c^***	**2.9**	3
E248C	−12.76 ± 3.20	4	**6.70 ± 0.24*^d^***	**0.2**	3	**10**	5.88 ± 0.24	1.3	4
K249C	−11.87 ± 1.86	4	6.10 ± 0.17	0.7	6	10	**5.60 ± 0.13*^d^***	**2.5**	7
L279C	−12.59 ± 1.87	8	6.30 ± 0.20	0.5	3	10	5.98 ± 0.36	1.1	5
N284C	−11.95 ± 4.19	7	**5.64 ± 0.06*^a^***	**2.3**	3	10	6.34 ± 0.13	0.5	5

*^a^ p* < 0.05.

*^b^ p* < 0.0001.

*^c^ p* < 0.01.

*^d^ p* < 0.001.

### Molecular docking of PPADS provides a range of potential binding poses

The data for the K249C mutant demonstrated that this residue forms part of the PPADS-binding site, and provided an anchoring point for ligand docking studies to predict potential PPADS binding modes. We therefore centered the docking site at Lys-249 (Fig. 1). To optimize conformational sampling in the ligand docking process, apo and open forms of hP2X1R homology models were used as receptor structures. This gave rise to four top-ranked docking clusters with similar scores, located in proximity of Lys-249 and pointing toward the orthosteric site (Fig. 1). These clusters shared the overall binding site, but varied in positioning and relative orientations of PPADS/hP2X1R. A common feature of the clusters were interactions between the negatively charged sulfonate and phosphate groups of PPADS with positively charged side chains of the receptor. Due to the variety of positively charged residues within and in proximity of the ATP-binding site more than one of these poses seemed plausible. To go beyond ranking these poses based on docking scores alone (and its uncertainties) additional experiments were required.

### Biochemical validation of PPADS docked poses

Molecular docking explored the area around Lys-249 and identified a range of potential binding poses for PPADS with a strong preference for the location between Lys-249 and the ATP-binding site. To map the location of the PPADS-binding site experimentally we determined whether PPADS had any effect on the accessibility of a range of cysteine mutants on the receptor surface within the circle centered on residue 249 (radius 13 Å). This included residues around the perimeter of the circle opposite the orthosteric site that had been sampled in the docking studies to provide unbiased testing of predictions of the binding pose. We have previously shown 13 of these mutants to be accessible (14, 16, 17) and have produced an additional 9 new mutants (K138C, R139C, D170C, S175C, R203C, Q237C, E248C, K249C, and L279C, for properties see Table 1). The area defined by the circle can be divided in two with one-half corresponding to the ATP-binding pocket and the second adjacent to the orthosteric site.

As a control we tested whether PPADS had any effect on biotinylation of cysteine mutants outside the predicted area at the apex of the receptor (G301C), in the lower body (P196C), and at the membrane interface (G54C). Biotinylation at these three mutants was unaffected by PPADS treatment (Fig. 2). This demonstrates that PPADS does not inhibit the chemical reaction of MTSEA-biotin binding to a cysteine residue and that when PPADS does reduce biotinylation (*e.g.* at K249C) this results from reduced accessibility of the cysteine residue.

At the orthosteric site the first β strand (β1) of the extracellular loop contributes to the ATP-binding pocket (15). We have previously shown that alternate cysteine mutants on this strand corresponding to side chains facing the agonist pocket were biotinylated (18). We have now extended this work and shown that accessibility at positions S64C, S66C, K68C, and K70C on the β1 strand was significantly reduced in the presence of PPADS (Fig. 2). Biotinylation was also reduced at D170C (above the ATP pocket), A182C (close to the head region), K190C (lower body region), and L279C (at the interface of the ATP pocket and the left flipper). Biotinylation was unaffected by PPADS at R203C (lower extent of potential PPADS site of action), N284C and G288C (at the tip and connection of the left flipper to the body). At the CRH biotinylation was reduced for G123C and R139C. There was a low level of biotinylation at the CRH mutant K138C in the apyrase condition consistent with the limited access of this residue predicted from the hP2X1R homology model. However, there was a striking ∼8-fold increase in biotinylation following PPADS treatment (Fig. 2) demonstrating that binding of the antagonist induces a conformational change at the CRH region to increase access to position 138. These results indicate that PPADS binding results in significant changes in accessibility at/around the orthosteric ATP-binding site.

PPADS had no effect on the level of biotinylation at mutants Q237C, S175C, Q76C, and P78C that are on the opposite side of the potential PPADS-binding pocket from the ATP-binding site. However, biotinylation was reduced at V74C, T75C, and L77C. These are close/linked to the β1 strand body region of the ATP-binding site (Lys-68 and Lys-70).

From our biotinylation studies we are clearly able to identify a number of cysteine mutants where accessibility was reduced by PPADS. When plotted onto the homology model the majority of the residues encompass the region around the orthosteric ATP-binding site in agreement with the main clusters from PPADS ligand docking. However, the biotinylation experiments also imply other residues and the total area covered by residues showing changes goes beyond the size of a single PPADS molecule (Fig. 2). This suggests that either PPADS may bind at multiple partially overlapping sites on the receptor, or that PPADS binding in addition to directly blocking access to particular residues also causes conformational changes in the receptor that affects accessibility of residues not directly lining the binding site. Ligand-induced conformational change in the extracellular loop has previously been detected. ATP-binding induced decreases in cysteine mutant MTSEA-biotinylation at residues outside of the agonist-binding pocket (18), consistent with the ATP-bound crystal structure (15).

### Do mutants with PPADS-dependent changes in cysteine accessibility modify antagonist sensitivity?

To discriminate between cysteine mutants where the reduction in biotinylation results from the residue contributing directly to PPADS binding from those where it could result indirectly from a conformational change outside the antagonist-binding site we tested whether the mutation had an effect on the ability of PPADS to inhibit ATP evoked currents (Table 1, Fig. 3). We reasoned that mutations of residues directly involved in PPADS binding would have an effect on antagonist action, whereas those that resulted from a conformational change outside of the PPADS site would be unaffected. Identification of such key residues would also help to distinguish between different ligand-docking clusters. To standardize comparisons we determined the ability of PPADS to inhibit the response to an EC_90_ concentration of ATP (Table 1) (19). Within and around the orthosteric pocket PPADS sensitivity was unchanged for mutants, S64C, S66C, G123C, R139C, and L279C (we have previously shown that PPADS sensitivity is unaffected at K68C) (17). The apparent affinity of PPADS for the mutants K70C, K190C, and K249C was decreased ∼3-fold (from 0.8 to 2.3, 2.9, and 2.5 μm respectively). Interestingly all these mutants remove a positive charge from the surface of the orthosteric pocket suggesting that electrostatic interactions with the negatively charged PPADS have been disrupted. In support of this, removal of the negative charge in the pocket (D170C) increased sensitivity to PPADS 4-fold (from 0.8 to 0.2 μm). PPADS sensitivity at mutants outside the orthosteric pocket (A74C, T75C, L77C, A192C, and E248C) was unaffected by the individual cysteine mutants. The level of MTSEA-biotinylation was ∼8-fold higher following PPADS binding at the CRH mutant K138C. Interestingly PPADS affinity was reduced ∼3-fold for this mutant indicating that the positive charge contributed to the conformational rearrangement associated with binding of the antagonist.

At mutants where there was no effect of PPADS on MTSEA-biotin access there was the potential that this was because the mutation abolished binding of the antagonist. At positions 175, 203, 284, and 237 cysteine mutation had no effect on PPADS sensitivity (Table 2). The R203C mutant showed an ∼3-fold decrease in PPADS sensitivity (from 0.8 to 2.5 μm). A similar decrease in PPADS sensitivity was seen at K249C but this was associated with an ∼90% decrease in biotinylation. This suggests that Arg-203 does not directly line the PPADS-binding site but that the positive charge may contribute to the electrostatic pull toward the orthosteric site. We have previously shown the cysteine mutants Q76C and P78C have no effect on PPADS sensitivity (17). These results show that the lack of reduction of biotinylation by PPADS at these cysteine mutants cannot be accounted for by an effect on antagonist sensitivity and so rules them out as lining the PPADS-binding pocket.

**Table 2:** **PPADS sensitivity at cysteine mutants with no change in biotinylation following PPADS treatment**

Mutant	pIC_50_	IC_50_
		μ*m*
WT	6.12 ± 0.15	0.8
S175C	6.02 ± 0.08	0.9
**R203C**	**5.61 ± 0.20*^a^***	**2.5**
Q237C	6.19 ± 0.07	0.7
N284C	6.32 ± 0.13	0.5
G288C	6.37 ± 0.05	0.4

*^a^* Data are shown as mean ± S.D., *p* < 0.01, *n* = 5 for WT and 3 for the mutants.

By determining whether cysteine mutation had any effect on PPADS sensitivity we have been able to discriminate residues that showed a decrease in accessibility on PPADS binding into (i) those where PPADS sensitivity was unaffected and therefore not likely to contribute directly to antagonist binding and changes in accessibility are likely to result from antagonist-induced conformational change and (ii) residues around the orthosteric pocket where there was a change in sensitivity to PPADS indicating a direct effect of those residues on antagonist action. Plotting the cysteine mutants in the second group predicted to be involved in PPADS binding (K70C, D170C, K190C and K249C) on the hP2X1R homology model highlights a region around the orthosteric site of equivalent dimensions to the antagonist (Fig. 3).

### PPADS binding quenches fluorescence at MTS-TAMRA-labeled cysteine mutants in the antagonist-binding pocket

**Figure 4.**
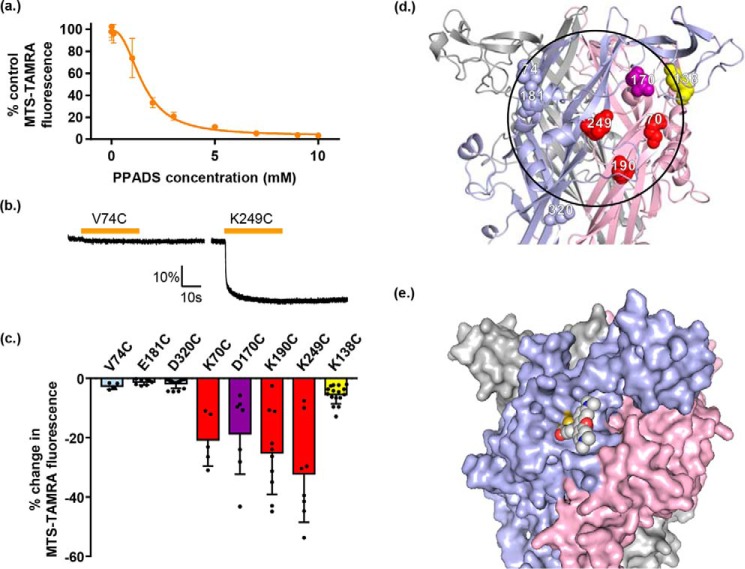
**PPADS quenches MTS-TAMRA fluorescence and highlights cysteine mutants lining the antagonist-binding site.** *a*, graph showing percentage of MTS-TAMRA fluorescence remaining after addition of different PPADS concentrations. The baseline fluorescence of 1 μm MTS-TAMRA was measured via Flexstation, then different concentrations of PPADS were added after 20 s and any change in fluorescence was monitored, data are shown as mean ± S.D. (*n* = 3). *b*, example traces of fluorometry recordings from oocytes expressing hP2X1R single cysteine point mutants labeled with MTS-TAMRA. Oocytes were perfused with ND96, then 10 μm PPADS was applied for 30 s by perfusion (*bar above trace*) and any changes in fluorescent output were measured. Changes in fluorescence were quantified as the percentage change in fluorescent output compared with baseline level measured before PPADS application. *Scale bars* apply to all traces. *c*, graph showing the average change in fluorescence for the mutants tested. A 5% decrease in fluorescence is shown as a *dotted line*, data are shown as mean ± S.D. (*n* ≥ 5). The effects of PPADS on cysteine accessibility and antagonist sensitivity were: V74C, E181C, and D320C (*light blue*) PPADS decreased in MTSEA-biotin and there was no change in PPADS sensitivity, at K70C, K190C, and K249C (*red*) MTSEA-biotin labeling was reduced following PPADS treatment and PPADS sensitivity was also reduced, at D170C (*purple*) PPADS decreased MTSEA-biotin access and sensitivity was increased, at K138C (*yellow*) PPADS increased MTSEA-biotin access and decreased PPADS sensitivity. *d*, hP2X1R homology model showing the positions of the introduced single cysteine residue mutations. *Blue* labeling indicates cysteine mutants with a PPADS induced decrease in MTSEA-biotinylation but no effect on PPADS sensitivity, *red* is for those with a decrease in MTSEA-biotinylation following PPADS treatment and a decrease in PPADS sensitivity, and *yellow* corresponds to residues where MTSEA-biotinylation was increased by PPADS and also showed a decrease in PPADS sensitivity. *e*, hP2X1R homology model surface representation with K249C linked MTS-TAMRA (modeled manually). Compared with *d,* the receptor is slightly rotated to the left for clearer visualization of MTS-TAMRA (shown as *spheres*).

Fluorescent probes can be attached to cysteine residues introduced into the extracellular loop of P2X1Rs and changes in fluorescence on ligand application have been used to understand drug binding (20–22). We therefore tested whether changes in fluorescence of labeled cysteine residues could be used to look at PPADS binding. We first tested whether PPADS had any effect on the fluorescence of the dye MTS-TAMRA that we have used previously to label hP2X1R cysteine mutants (21). Control studies showed that 10 μm PPADS (a concentration that inhibited WT hP2X1R currents by ∼95%) had no effect on the fluorescence of a 1 μm MTS-TAMRA solution (the concentration used to label the cysteine mutants) demonstrating that there is no interference between these compounds at these concentrations when mixed in solution. However, increasing the PPADS concentration (increasing the probability of PPADS and MTS-TAMRA being in close proximity) resulted in a concentration-dependent quenching of fluorescence (50% decrease at ∼2 mm)(Fig. 4). Thus if a MTS-TAMRA–labeled cysteine is close to the PPADS-binding site we would predict that binding of PPADS would decrease MTS-TAMRA fluorescence. We have previously shown that D320C (lower body outside of the area defined by the radius of PPADS centered on Lys-249) can be labeled with MTS-TAMRA and fluorescence detected (21). PPADS (10 μm) application had no effect on fluorescence at MTS-TAMRA-labeled D320C. MTS-TARMA fluorescence at residues V74C and E181C was also not affected by PPADS (Fig. 4) demonstrating that these residues do not come into close proximity with the antagonist. We set a detection threshold for a PPADS effect at 5% fluorescence change. For the mutants that showed a change in PPADS sensitivity and reduction in MTSEA-biotin access, indicative of being part of the binding pocket (K70C, D170C, K190C, and K249C) MTS-TAMRA fluorescence was reduced significantly by 20–30% following binding of the antagonist. At residue K138C (increased access following binding of the antagonist) MTS-TAMRA fluorescence was also decreased by PPADS suggesting that the base of the head region moves into closer proximity to the antagonist-binding site. These results where MTS-TAMRA fluorescence was reduced, taken together with the biotinylation and antagonist sensitivity data highlight the region defined by Lys-249, Asp-170, Lys-70, and Lys-190 as the site of PPADS binding.

### Revisiting molecular docking of PPADS in context of cysteine mutagenesis mapping

**Figure 5.**
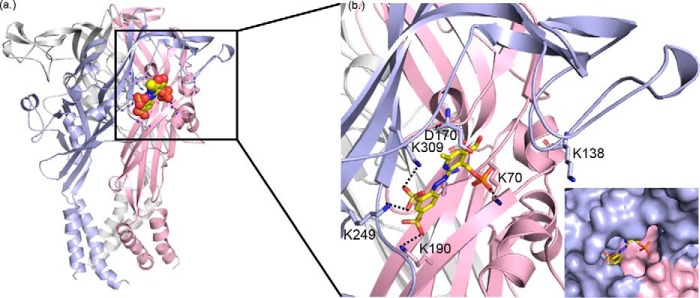
**PPADS docking pose in best agreement with experimental data.** *a*, *cartoon* representation of open state hP2XR1 model with subunits shown in *red*, *blue*, and *white*, respectively. The docked PPADS pose from the largest, top ranked cluster that is best explaining data for Lys-70, Asp-170, Lys-190, and Lys-249 is shown as *spheres. b,* zoom into *a*, detailing key charge interactions between PPADS and hP2XR that are discussed in the text.

Integrating docking results with the measured changes in accessibility of individual residues upon PPADS binding, the effect of mutations on PPADS potency, and defining the overall location of the PPADS-binding site from MTS-TAMRA fluorescence quenching experiments, allowed us to determine docking poses that agree best with the information gathered from experiments. The reduction in PPADS potency and loss of accessibility upon PPADS binding suggest that Lys-70, Lys-190, and Lys-249 are directly involved in PPADS binding. This favors a PPADS-binding site partially overlapping with the binding site for ATP, and is best reflected in a representative pose of the largest, first ranked cluster from PPADS ligand docking to the hP2X1R model in the open state. In this binding pose the PPADS-phosphate group forms a salt bridge with Lys-70 and Lys-68, and the two PPADS-sulfonate groups bridge two subunits of P2X1R and form salt bridges with Lys-190 and Lys-249/Lys-309 (Fig. 5). The proposed binding mode suggests binding of PPADS to all three orthosteric sites. This is consistent with the Hill slope of 1.4 in the inhibition curve of the antagonist consistent with more than one molecule of PPADS binding required to block ATP action.

## Discussion

In this study we used cysteine mutagenesis to map the binding site for the P2XR antagonist PPADS. If a residue was directly involved in PPADS binding we predicted that it would show three properties: (i) PPADS binding would decrease accessibility of the introduced cysteine residue (measured by MTSEA-biotinylation), (ii) the cysteine mutant would have an effect on PPADS sensitivity, and (iii) PPADS binding would quench MTS-TAMRA fluorescence. These criteria were met by four (Lys-70, Asp-170, Lys-190, and Lys-249) of 26 residues tested and when mapped onto the hP2X1R they define an area at the orthosteric binding pocket equivalent to that of the PPADS molecule. Combining these data with molecular docking enabled us to produce a model of the antagonist-binding site. PPADS binding overlaps with the orthosteric ATP site and so binding of the antagonist would sterically block access to the agonist. We also demonstrated binding of the antagonist evoked significant conformational change within the head region of hP2X1R.

The contribution of lysine 249 to PPADS binding was originally suggested from work on the rP2X4R (7). In the current study we confirm this finding by showing that access to, and MTS-TAMRA fluorescence at, the hP2X1R K249C was reduced by PPADS. In addition the K249C mutant showed reduced sensitivity to the antagonist. These three lines of evidence strongly support that this residue is directly involved in the binding of PPADS to the hP2X1R. The rP2X4R cloning paper reported that the E249K mutation changed the receptor from one that was essentially insensitive to the antagonist (IC_50_ > 500 μm) to one where PPADS had an IC_50_ of ∼10 μm (7) raising the possibility that positive charge at this position played a dominant role in determining sensitivity to the antagonist. However, additional experiments showed that the relative contribution of the lysine at position 249 (P2X1 and -4 numbering) depended on the species and background. The hP2X4R (that also has a glutamic acid at position 249) has an IC_50_ of ∼30 μm (8). The “reverse” Lys–Glu charge mutation at the rP2X2R had no effect on PPADS sensitivity (7), and the equivalent residue in the P2X3R that has a PPADS IC_50_ of 1–5 μm (1) is not charged. In the current study on the hP2X1R there was a modest ∼3-fold decrease in sensitivity at the K249C mutant. These results demonstrate that the positive charge at position 249 can contribute to the action of PPADS but that other residues must also be involved.

**Figure 6.**
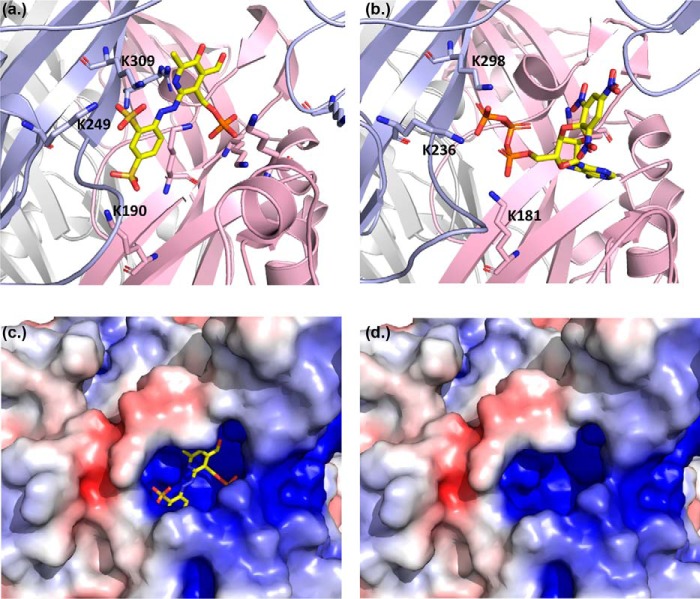
**Comparison of proposed PPADS and TNP-ATP binding modes.** *a*, proposed PPADS binding mode for P2X1R. *b*, for comparison the X-ray structure of chicken P2X7 receptor with TNP-ATP bound is shown. Equivalent lysine residues involved in salt bridges to sulfonate groups of PPADS (*a*) and phosphate groups of TNP-ATP are labeled. *c*, visualization of electrostatic potential for the P2X1R model used in *a*. The electrostatic potential was calculated using the Protein Data Bank 2PQR. *d,* as in *c*, but PPADS was omitted.

One characteristic of ATP binding to P2X receptors is the interaction between positively charged residues on the receptor and the negatively charged triphosphate moiety of ATP. Like ATP, PPADS is a highly negatively charged compound. In this respect one would expect (as for ATP) that one of the driving forces for PPADS binding is the electrostatic pull by positively charged residues in and around the ATP-binding site (Fig. 6), and that the binding mode is characterized by specific charge-charge interactions. So it is perhaps not surprising that in addition to Lys-249 the conserved residues involved in ATP binding Lys-70, Lys-190, and Lys-309 are predicted to directly bind to PPADS in our preferred model of antagonist binding. The binding pose best supported by experimental data overlaps with the ATP-binding site and shows PPADS forming salt bridges with Lys-70, Lys-190, and Lys-249, residues where mutation to cysteine resulted in a significant change in antagonist potency. The binding mode also comprises additional charged interactions with residues known to coordinate the triphosphate group of ATP (Lys-68 and Lys-309), indicating that blocking ATP from binding may be part of the PPADS mode of action. The reduction of accessibility found for Ser-64, Ser-66, and Lys-68 is consistent with PPADS bracing adjacent subunits of hP2X1R via salt bridges between sulfonate groups and the side chains of Lys-190 and Lys-249, and therefore blocking access to the deeper reaches of the pocket where Ser-64, Ser-66, and Lys-68 are located. Furthermore, there are positively charged residues in the proximity of the proposed PPADS-binding site that do not interact directly with PPADS in the docked pose. For instance, the lysine on the dorsal fin (Lys-215 in the hP2X1R) that is conserved on P2X1–6Rs may also contribute to the electrostatic pull to the pocket (at the K215C mutant PPADS sensitivity and MTS-TAMRA fluorescence were reduced ∼3-fold and ∼15%, respectively).^3^ Interestingly there is a deletion for the P2X7R removing 4 residues in the dorsal fin suggesting that it is not essential for PPADS binding (23).

The importance of the electrostatic environment around the PAPDS-binding pocket is further supported by the ∼3-fold increased PPADS sensitivity following removal of the negative charge at position 170 in the hP2X1R. The proposed binding mode allows rationalizing the effect of the D170C mutation on PPADS potency. A negative charge is also found at the equivalent position in hP2X3 and -4Rs, there is no charge at P2X2, -6, and -7 receptors, and a positive charge at the rP2X5R. This suggests that variations at this position may contribute to subtype-dependent differences in PPADS sensitivity. Overall the modest ∼3-fold change in sensitivity for mutants predicted to directly interact with PPADS is consistent with a set of positively charged residues required for PPADS binding, but with no particular residue exerting a dominating effect at the hP2X1R. Taken together our results suggest that the local electrostatic environment has a large role to play in action and can be “compensated” in different ways.

**Figure 7.**
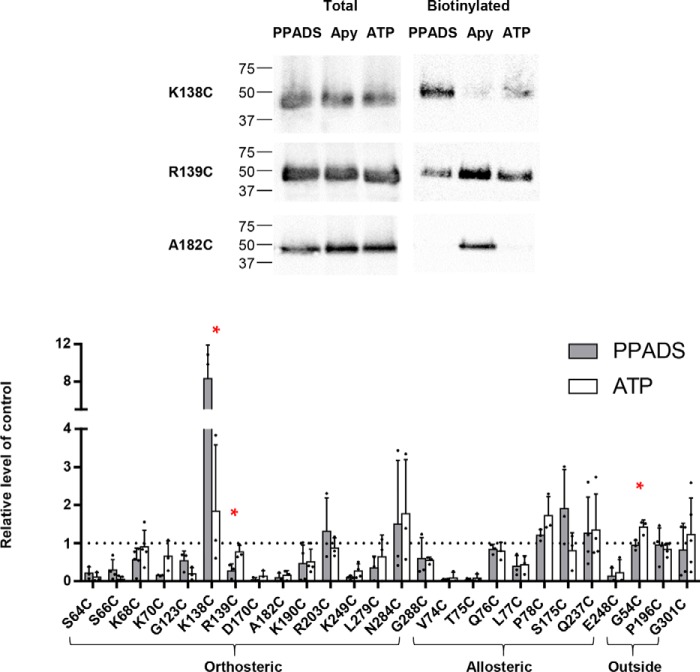
**Comparison of the effects of PPADS and ATP on MTSEA-biotinylation at cysteine mutants.** *Upper panel* shows representative blots of the MTSEA-biotinylation levels of mutant P2X1Rs in the presence of PPADS, apyrase control, or ATP. The graph shows the effects of PPADS (*gray* shading) or ATP (*open boxes*) on the relative level of biotinylation compared with the control (in the presence of apyrase to break down any endogenous ATP). Data are shown as mean ± S.D. (*n* = 3), *, *p* < 0.05.

**Figure 8.**
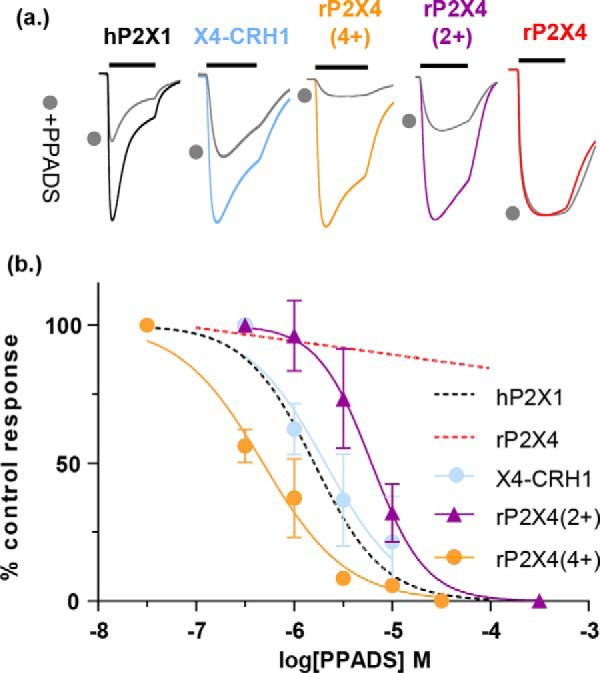
**Positive charge at the base of the cysteine-rich head region imparts PPADS sensitivity to the rP2X4R.** *a,* representative traces showing application of ATP and ATP + 1 μm PPADS (*filled circles*) for rP2X4R, the X4-CRH1 chimera and hP2X1R for rP2X4(2+) and rP2X4(4+) mutant receptors. *b,* PPADS inhibition curves. All inhibition was measured at an EC_90_ concentration of ATP. *Dotted lines* correspond to previously published mean data from Farmer *et al.* (19). The *black bar* corresponds to the period of agonist application (3 s) and PPADS was pre-superfused over the oocyte and co-applied with ATP (*filled circles*). Traces have been normalized to peak current to allow for comparison. Data are plotted as mean ± S.D. *n* = 3–4.

Ligand binding stabilizes a particular receptor structure and can be associated with conformational change from the apo state. Our study shows that in addition to reductions in MTSEA-biotin labeling consistent with PPADS directly blocking access the antagonist evoked marked changes in MTSEA-biotin access at residues outside of the predicted binding site. These highlight that PPADS binding induces conformational movement around the orthosteric pocket from the closed apo state. For the majority of mutants not directly at the PPADS-binding site that showed a decrease in access to the antagonist, a similar decrease in access was seen for ATP (Fig. 7). Interestingly at the hP2X1R there was marked variation in the effects of PPADS and ATP on the level of biotinylation for K138C with ∼8-fold increases seen with PPADS and essentially no change with ATP and at the adjacent residue and R139C an ∼4-fold decrease with PPADS but no effect for ATP (Fig. 8). These results suggest that PPADS binding results in a distinct conformational state that is more similar to the ATP-bound than the closed state. A similar move toward the ATP-bound state has been recently reported for the antagonist action of TNP-ATP at the chicken (ch) P2X7R. Co-crystallization of the chP2X7R with TNP-ATP showed an incompletely activated conformation that was closer to the ATP bound than the apo state (24). The PPADS binding mode for hP2X1R described above shows aspects remarkably similar to the TNP-ATP binding mode in chP2X7R. For instance, the two sulfonate groups are in equivalent positions and coordination environments to α- and γ-phosphates in TNP-ATP with residues Lys-309, Lys-249, and Lys-190 in hP2X1, and Lys-298, Lys-236, and Lys-181 in chP2X7 forming salt bridges to the antagonists (Fig. 6). Compared with the chP2X7R the binding mode for TNP-ATP proposed for hP2X3R shows a different conformation with the receptor essentially in the apo state (11). This indicates that alternative binding modes for the same antagonist may be possible in different P2XR subtypes. In the context of PPADS binding, this suggests that there may be more than one conformation or PPADS binding mode contributing to potency and differences in subtype specificity.

Our study showed that PPADS-induced movement of the CRH is distinct from that for ATP and raises the possibility that this region contributes to the action of the antagonist. This is supported by four lines of evidence: (i) we previously showed a chimera replacing the CRH region of the P2X1R with that from the rP2X4R reduced PPADS sensitivity (19), (ii) mutation of the P2X7R to remove a positively charged arginine residue at the base of the CRH (R126G) reduced PPADS affinity ∼10-fold (9), (iii) a chimera based study to determine the differences in PPADS sensitivity between rat and human P2X4Rs showed that a region including the CRH was involved in determining sensitivity to the antagonist (8), and (iv) in this study we show that the K138C mutant had reduced PPADS sensitivity. In the model, based on the open state, Lys-138 is in proximity of the PPADS-binding site (but not directly interacting with the antagonist) and is contributing to the overall positively charged environment that is pulling the negatively charged PPADS toward the hP2X1 receptor. To test directly whether variation in the CRH contributed to the lack of effect of PPADS at the rP2X4R we generated the reciprocal chimera replacing the CRH head region (residues 133–184) of the rP2X4R with that from the hP2X1R (chimera named X4-CRH1). In support of the hypothesis the PPADS sensitivity of the X4-CRH1 chimera was indistinguishable from the hP2X1R (Fig. 8). At the base of the CRH of the hP2X1R are four positively charged residues (Lys-136, Lys-138, Arg-139, and Lys-140). One of these, lysine 138 is an aspartic acid in the rP2X4R and a glycine in the hP2X4R raising the possibility that negative charge at the base of the CRH contributes to the lack of PPADS inhibition at the rP2X4R. To determine whether the introduction of positive charge at the base of the CRH accounted for the acquired PPADS sensitivity of chimera X4-CRH1, two further rP2X4R-based mutants were generated. The first, rP2X4(2+), had two point mutations, S136K and D138K. The second, rP2X4(4+), had these mutations and a further two mutations T139R and H140K and was equivalent to the charge at the hP2X1R. The mutants increased PPADS sensitivity to within ∼3-fold of the hP2X1R. These results highlight that charge and conformational change around the CRH region contribute to PPADS binding.

In this study we have provided an experimentally validated model of the binding site of the P2XR antagonist PPADS. This involves interaction of a range of charged residues with the antagonist. Many of these core residues are conserved between P2XR subunits. The ∼3-fold changes in sensitivity for individual mutants in the current study provide a template for understanding modest differences in sensitivity between P2X1–3, -5, and -6Rs. Before information on the structure of P2XRs became available the Jacobson group (25) synthesized iso-PPADS derivatives that showed that improved subtype selectivity. Mechanistic understanding of the site of PPADS action and the underlying subtype variations in this study provide the potential of medicinal chemistry to develop more subtype-selective orthosteric antagonists. Our study has identified core conserved residues in the orthosteric pocket that coordinate PPADS binding and in addition show that interaction with the CRH plays a significant role. Analysis of the different P2XR subunits shows that there is significant variation in the sequence at the base of the cysteine-rich head region. Our results highlight that this variation could potentially be exploited to develop subtype-selective antagonists. We propose that a family of compounds could be developed that have a core that is compatible with the orthosteric site (but that on its own has low affinity) and subtype selectivity is imparted by specific side group substitutions that have high affinity for variant residues at the base of the CRH found in the different P2XR subtypes.

## Experimental procedures

### Molecular biology

The hP2X1 receptor cDNA was originally cloned from the bladder (23) and the rP2X4 receptor DNA was a gift of Dr. Francois Rassendren (CNRS, Montpellier, France). A mutation (Y378A) was introduced to the rP2X4 receptor template to give more stable and reproducible currents (24). Point mutations were made using the QuikChange mutagenesis kit (Stratagene). Mutations were verified by DNA sequencing by the Protein and Nucleic Acid Chemistry Laboratory services, University of Leicester.

### Expression in Xenopus laevis oocytes

cRNA was synthesized using the T7 mMessage mMachine kit (Ambion) and injected into stage V *X. laevis* oocytes as described previously (23). Oocytes expressing P2X receptors were stored at 16 °C in ND96 buffer (96 mm NaCl, 2 mm KCl, 1.8 mm CaCl_2_, 1 mm MgCl_2_, 5 mm sodium pyruvate, 5 mm HEPES, pH 7.6) with 50 μg ml^−1^ of gentamycin and tetracycline for 3–7 days. For electrophysiological recordings antibiotics were not present and the 1.8 mm CaCl_2_ was replaced with 1.8 mm BaCl_2_.

### MTSEA-biotinylation

Oocytes injected with WT or mutant receptor RNA were divided into two equivalent groups (7–10 oocytes in each group) labeled as apyrase and PPADS groups, respectively. Oocytes in the apyrase group were preincubated in 0.5 ml of ND96 with 15 units/ml of apyrase (Sigma, Poole, UK) and those in the PPADS group were preincubated in ND96 with 100 μm PPADS (Cayman, Ann Arbor, MI) for 10 min. 100 μm MTSEA-biotin (BIOTIUM, Cambridge, UK) was applied to the oocytes in the two conditions for 90 s. After the oocytes were washed 3 times with the preincubation solutions, they were placed in 1.5-ml tubes and the excess liquid was removed. Oocytes were lysed with 100 μl buffer H (100 mm NaCl, 20 mm Tris Cl, 1% Triton X-100, pH 7.4, 10 μl/ml of proteinase inhibitor mixture was added before use) per tube and pipetting with a 200-μl pipette. After vortexing, the samples were left on ice for 10 min and then were centrifuged at 4 °C for 5 min at 13,000 rpm. The supernatant samples were pipetted to new tubes and 15 μl was removed, to act as the total sample, and placed in the freezer. 200 μl of buffer H and 50 μl of streptavidin-agarose beads (Life Technologies, Paisley, UK) were added to the remaining samples labeled as biotin sample and were rolled at 4 °C overnight to allow the protein bound by MTSEA-biotin to bind to the beads. The beads were then washed 5 times with 0.5 ml of buffer H per wash and spun for 5 min between each wash to isolate the protein bound by MTSEA-biotin. Both total and biotin samples had 25 and 15 μl of SDS sample buffer (0.18 m Tris base, 5.7% SDS, 29% glycerol, 0.003% bromphenol blue, pH 6.8, 5% β-mercaptoethanol added immediately prior to use) added, respectively, and were heated at 95 °C for 5 min. Samples were vortexed and spun down for 5 min at 13,000 rpm and they were then loaded on gels. Finally, the total and biotinylated P2X1R proteins in apyrase and PPADS treatment were quantified by Western blotting with an anti-P2X1R antibody (1:2000) (APR-001 lot number AN0802, Alomone, Jerusalem, Israel) as described previously (18).

### Electrophysiological recordings

Two electrode voltage-clamp recordings were carried out using a Geneclamp 500B amplifier with a Digidata 1322A analogue-to-digital converter and pClamp 8.2 acquisition software (Molecular Devices, Menlo Park, CA) at a holding potential of −60 mV. ATP (Mg^2+^ salt, Sigma) was applied via a U-tube perfusion system for 3 s at 5–10–min intervals (dependent on the P2X receptor mutant) to allow for reproducible responses to be recorded (19). Antagonists were bath perfused in ND96 solution for 5 min before they were co-applied with an EC_90_ concentration of ATP through the U-tube. To generate inhibition curves antagonists were co-applied with an EC_90_ concentration of ATP to standardize any shift in ATP potency. Antagonists fully equilibrated with the receptor during the first application period as the level of inhibition was maintained on a second test application. The inhibitory effects of the antagonists were reversed in the washout period between agonist applications. Antagonists, at maximum concentrations used, were applied to all WT and mutant receptors in the absence of ATP and were seen to have no effect on the holding current. PPADS was from Tocris.

### MTS-TAMRA labeling and voltage-clamp fluorometry

Voltage-clamp fluorometry (VCF) recordings were undertaken as previously reported (21). Oocytes injected with cRNA were used for two-electrode voltage-clamp recordings using an Axoclamp 900A amplifier with a Digidata 1440A analog-to-digital converter and pClamp 10.2 acquisition software (Molecular Devices). VCF recordings were performed using a custom organ bath, designed to apply drug solutions directly to the underside of the oocyte. Drugs were applied using a ValveLink 8 perfusion system (AutoMate Scientific, Berkeley, CA) electronically controlled by the pClamp protocol to ensure rapid solution exchange. For imaging the oocytes, the organ bath was installed on a Nikon Diaphot 200-inverted microscope equipped with HQ545/30 exciter, Q565LP dichroic and HQ572LP emitter filter set, with OptoLED lite light source (Cairn Research, Faversham, UK). Fluorescence was detected by a photomultiplier tube (Cairn Research) installed to the side port of the microscope, with data recorded using pClamp 10.2. Oocytes were incubated in 5 μm MTS-TAMRA (Toronto Research Chemicals, Toronto, Canada) for 60 s, washed in excess ND96 and stored on ice in the dark prior to VCF recordings. During VCF recordings, the baseline fluorescence output of the oocyte was recorded for 10 s before drug application. Any change in observed fluorescence was calculated as the percentage change from this pre-application level.

In control studies, measuring fluorescence using a Flexstation II plate reader (Molecular devices), PPADS (up to 10 mm), was added to 1 μm MTS-TAMRA after 20 s. Any change in fluorescence output was calculated as the percentage change against the baseline level.

### Modeling/docking

Ensembles of 100 structural models for hP2X1R in the closed and open state were generated in Modeler 9v14 (28) using zfP2X4R structures as templates (PDB identifiers 4DW0 and 4DW1) (15). Ten representative hP2X1R models for each state were selected as receptors for ligand docking. PPADS was docked into the hP2X1R models using RosettaLigand (26), the docking area was centered at Lys-249. For both states 10,000 docking poses were generated. Of these the best scoring 10% docked complexes were clustered using cpptraj (27). From the resulting clusters representative poses were visualized in PyMol and further analyzed in the context of the experimental data.

### Data analysis

MTSEA-biotinylation between apyrase and PPADS/ATP treatment was measured by densitometry (ImageJ) and corrected for any differences in the total amount of P2X1R protein in the two samples and expressed as the level of biotinylation for drug treatment relative to the apyrase control. Any differences in labeling between apyrase and apyrase + drug treatment were compared with paired Student's *t* test.

Individual normalized concentration-response curves were fitted with the Hill equation (variable slope) with GraphPad Prism 6. For agonists pEC_50_ is the -log_10_ of the concentration giving 50% maximal response (EC_50_ value), and IC_50_ is the concentration of antagonist inhibiting the EC_90_ concentration of ATP by 50%. pIC_50_ is the -log_10_ of the IC_50_ value. For the calculation of EC_50_/IC_50_ values and Hill slopes, individual concentration-response curves were generated for each experiment and statistical analysis was carried out on the data generated. In the figures, inhibition curves are fitted to the mean normalized data. Data are shown as mean ± S.D. Any significant differences in ligand sensitivity were calculated by one-way analysis of variance followed by Dunnett's test. The software used was GraphPad Prism 6 (GraphPad Software Inc., San Diego, CA). Data are shown as mean ± S.D., *n* ≥ 3 for all data points.

## Author contributions

H. H., A. G. F., L. K. F., and R. S. formal analysis; H. H., L. K. F., and R. S. investigation; H. H., A. G. F., L. K. F., R. S., and R. J. E. writing-review and editing; A. G. F. and R. J. E. conceptualization; A. G. F., R. S., and R. J. E. writing-original draft; R. J. E. supervision; R. J. E. funding acquisition; R. J. E. project administration.

## Footnotes

3 A. G. Fryatt and R. J. Evans, unpublished observations.

## Footnotes

2 The abbreviations used are:

P2XR: P2X receptors
PPADS: pyridoxalphosphate-6-azophenyl-2′,4′-disulfonic acid
CHR: cysteine-rich head
MTSEA: 2-((6-((6-((biotinoyl)amino)hexanoyl)amino)hexanoyl)amino)ethyl methanethiosulfonate
TAMRA: 2-((5(6)-tetramethylrhodamine)carboxylamino)ethyl
TNP-ATP: 2′(3′)-*O*-(2,4,6-trinitrophenyl)adenosine 5′-triphosphate
VCF: voltage-clamp fluorometry.
